# Treatment Discontinuation of First-Line Bosutinib in a Real-World Cohort of Patients With Chronic Myeloid Leukemia: Impact of Hepatic Toxicity

**DOI:** 10.7759/cureus.108385

**Published:** 2026-05-06

**Authors:** Masuho Saburi, Kuniaki Maehara, Takumi Nishikawa, Takata Hiroyuki, Masanori Sakata, Kazuhiro Kohno, Yuko Ogata, Takami Haruyama, Eiichi Ohtsuka

**Affiliations:** 1 Department of Hematology, Oita Prefectural Hospital, Oita, JPN; 2 Hematology, Oita Kouseiren Tsurumi Hospital, Beppu, JPN; 3 Hematology, National Hospital Organization Beppu Medical Center, Beppu, JPN; 4 Hematology, Japan Community Health Care Organization (JCHO) Nankai Medical Center, Saiki, JPN

**Keywords:** bosutinib, chronic myeloid leukemia, hepatic toxicity, real-world study, treatment discontinuation

## Abstract

A multicenter, retrospective study to evaluate the efficacy and safety of first-line bosutinib in patients with newly diagnosed chronic myeloid leukemia (CML) in real-world clinical practice was conducted. Twenty-nine consecutive patients were included between August 2020 and October 2024. The primary endpoint was the cumulative incidence of major molecular response (MMR) at 12 months. The median age was 64 years, and most patients started at a reduced dose. The cumulative incidence of MMR at 12 months was 67.9%, and the overall MMR rate was 20/29 patients (69.0%). The cumulative incidence of MR4 at 12 months was 22.1%, with an overall MR4 rate of 14/29 patients (48.3%). The 24-month overall survival rate was 100%. Bosutinib was discontinued in 14/29 patients (48.3%), and the 24-month cumulative incidence of treatment discontinuation was 51.8%. Dose reduction occurred in 17/29 patients (58.6%) and treatment interruption in 14/29 patients (48.3%). Alanine aminotransferase elevation was the leading cause of discontinuation. First-line bosutinib achieved favorable molecular responses. However, treatment discontinuation due to hepatic toxicity remains a clinical concern in real-world practice.

## Introduction

Chronic myeloid leukemia (CML) is a myeloproliferative neoplasm characterized by the Philadelphia chromosome resulting from the reciprocal translocation t(9;22)(q34;q11), which generates the BCR::ABL1 fusion gene with constitutive tyrosine kinase activity. In the absence of effective therapy, CML progresses from chronic phase (CP) to accelerated phase (AP) and blast phase (BP), leading to poor outcomes [1-3]. The introduction of BCR::ABL1 tyrosine kinase inhibitors (TKIs) dramatically improved survival outcomes and established long-term disease control in patients with CML-CP [4]. Several second-generation TKIs, including nilotinib, dasatinib, and bosutinib, are recommended as first-line treatment options for newly diagnosed CML-CP, taking into account disease risk and patient comorbidities [5-7]. With the achievement of deep molecular responses, treatment goals have shifted from preventing disease progression to achieving sustained treatment-free remission [8]. Bosutinib was approved as first-line therapy based on the BFORE trial [7,9], which demonstrated superior molecular response rates than imatinib. A Japanese phase II study also reported good long-term molecular responses [10,11]. However, gastrointestinal and hepatic adverse events were frequently observed, and treatment discontinuation due to toxicity remains a clinical concern. In real-world clinical practice, patient characteristics often differ from those in clinical trials, particularly due to age, comorbidities, and performance status. Moreover, dose modifications and individualized starting doses are commonly used in real-world settings. Real-world outcomes on first-line bosutinib remain limited, especially about tolerability or treatment discontinuation. Therefore, a multicenter, retrospective study was conducted to evaluate the efficacy, survival outcomes, and safety profile of bosutinib as initial therapy for newly diagnosed CML-CP in real-world clinical practice.

## Materials and methods

Patients

This multicenter, retrospective study included consecutive patients with newly diagnosed CML-CP who received bosutinib as first-line therapy between August 2020 and October 2024 at four institutions in Oita Prefecture, Japan (Oita Prefectural Hospital, Oita Kouseiren Tsurumi Hospital, National Hospital Organization Beppu Medical Center, and Japan Community Health Care Organization (JCHO) Nankai Medical Center). Eligible patients were required to have newly diagnosed Philadelphia chromosome-positive and/or BCR::ABL1-positive chronic myeloid leukemia, and sufficient medical records for baseline characteristics, treatment course, response assessment, adverse events, and follow-up. Patients who had received prior tyrosine kinase inhibitor therapy or had blast phase disease at treatment initiation were excluded. Clinical data, including age, sex, performance status, comorbidities, laboratory findings at diagnosis, risk scores, treatment dose and modifications, response assessments, adverse events, and survival outcomes, were collected from medical records. The starting dose and subsequent dose adjustments of bosutinib, as well as the intervals of efficacy assessment, were not protocol-defined but were determined at the discretion of the treating physicians based on patient characteristics, treatment response including hematologic and molecular responses, and tolerability. This study was conducted in accordance with the Declaration of Helsinki and was approved by the ethics review board of Oita Prefectural Hospital (approval number 6-191). Patients’ informed consent was obtained in the form of opt-out on the website.

Methods, definitions, and statistical analysis

The primary endpoint was the cumulative incidence of major molecular response (MMR). Exploratory endpoints included rates of complete hematological response (CHR) and MR4, overall survival (OS), the cumulative incidence of treatment discontinuation, and adverse events (AEs). Baseline risk was assessed using the European Treatment and Outcome Study (EUTOS) long-term survival (ELTS) score, which stratifies patients into low-, intermediate-, and high-risk categories according to the long-term probability of dying from CML [12]. BCR::ABL1 transcript levels, measured using the International Scale (IS) [13], were monitored according to each physician’s clinical practice. Molecular responses were assessed according to the European Leukemia Net 2020 criteria [14]. MMR was defined as IS ≤0.1%, and MR4 as IS ≤0.01%. AEs were graded according to the Common Terminology Criteria for Adverse Events (CTCAE), version 5.0, from grade 1 (mild) to grade 5 (death related to an adverse event) [15]. OS was calculated from the start of bosutinib to death from any cause or last follow-up. The cumulative incidences of MMR, CHR, MR4, and treatment discontinuation were estimated using competing risk analysis. For molecular and hematological responses, treatment discontinuation, progression, or death before achieving each response was considered a competing event. For treatment discontinuation, death before discontinuation was considered a competing event. Survival curves were estimated using the Kaplan-Meier method. Statistical analyses were performed using EZR software [16].

## Results

Patient characteristics

Baseline characteristics are summarized in Table 1. A total of 29 patients with newly diagnosed CML were included. The median age at diagnosis was 64 (range, 17-84) years, and 18 patients (62%) were male. At treatment initiation, 27 patients were in CP, two were in AP. According to ELTS risk stratification, 15 patients (51.7%) were classified as low risk, 13 (44.8%) as intermediate risk, and one (3.4%) as high risk. Additional cytogenetic abnormalities were observed in five patients (17.2%). Comorbidities were common, including hypertension in 12 patients (41.4%), diabetes mellitus in 13 (44.8%), dyslipidemia in 12 (41.4%), and cardiovascular disease in five (17.2%). Three patients had a performance status ≥2 at treatment initiation. The starting dose of bosutinib is summarized in Table 1, with most patients beginning treatment at a reduced dose relative to the standard recommended dose of 400 mg.

**Table 1:** Patients’ characteristics (n=29). Continuous variables are expressed as median (range), and categorical variables as number (percentage). ELTS: European Treatment and Outcome Study (EUTOS) long-term survival score.

Characteristic	Value
Age, y	64 (17–84)
Male sex, n (%)	18 (62.1%)
Body weight, kg	61.8 (37.3–109.0)
Body surface area, m^2^	1.67 (1.26–2.30)
White blood cell count, ×10^9^/L	29.0 (7.7–40.5)
Hemoglobin, g/dL	13.1 (8.7–17.7)
Platelet count, ×10^9^/L	381 (134–1,396)
Lactate dehydrogenase, U/L	432 (189–2,846)
Disease phase at treatment initiation, n (%)	
Chronic phase	27 (93.1%)
Accelerated phase	2 (6.9%)
Blast phase	0 (0%)
ELTS risk category, n (%)	
Low	15 (51.7%)
Intermediate	13 (44.8%)
High	1 (3.4%)
Additional cytogenetic abnormalities, n (%)	5 (17.2%)
Comorbidities, n (%)	
Hypertension	12 (41.4%)
Diabetes mellitus	13 (44.8%)
Dyslipidemia	12 (41.4%)
Cardiovascular disease	5 (17.2%)
Performance status at treatment initiation, n (%)	
0	19 (65.5%)
1	7 (24.1%)
2	1 (3.4%)
3	2 (6.9%)
Starting dose of bosutinib, n (%)	
100 mg	10 (34.5%)
200 mg	14 (48.3%)
300 mg	1 (3.4%)
400 mg	4 (13.8%)

Treatment exposure

The maximum dose achieved was 200 mg in two patients (6.9%), 300 mg in five (17.2%), 400 mg in 21 (72.4%), and 500 mg in one (3.4%), with 22 patients (75.9%) eventually reaching a dose ≥400 mg during treatment. Dose reduction was required in 17 patients (58.6%), and treatment interruption occurred in 14 (48.3%). The final continuous dose was 200 mg in 12 patients (41.4%), 300 mg in five (17.2%), and 400 mg in 11 (37.9%).

Response and survival

**Figure 1:**
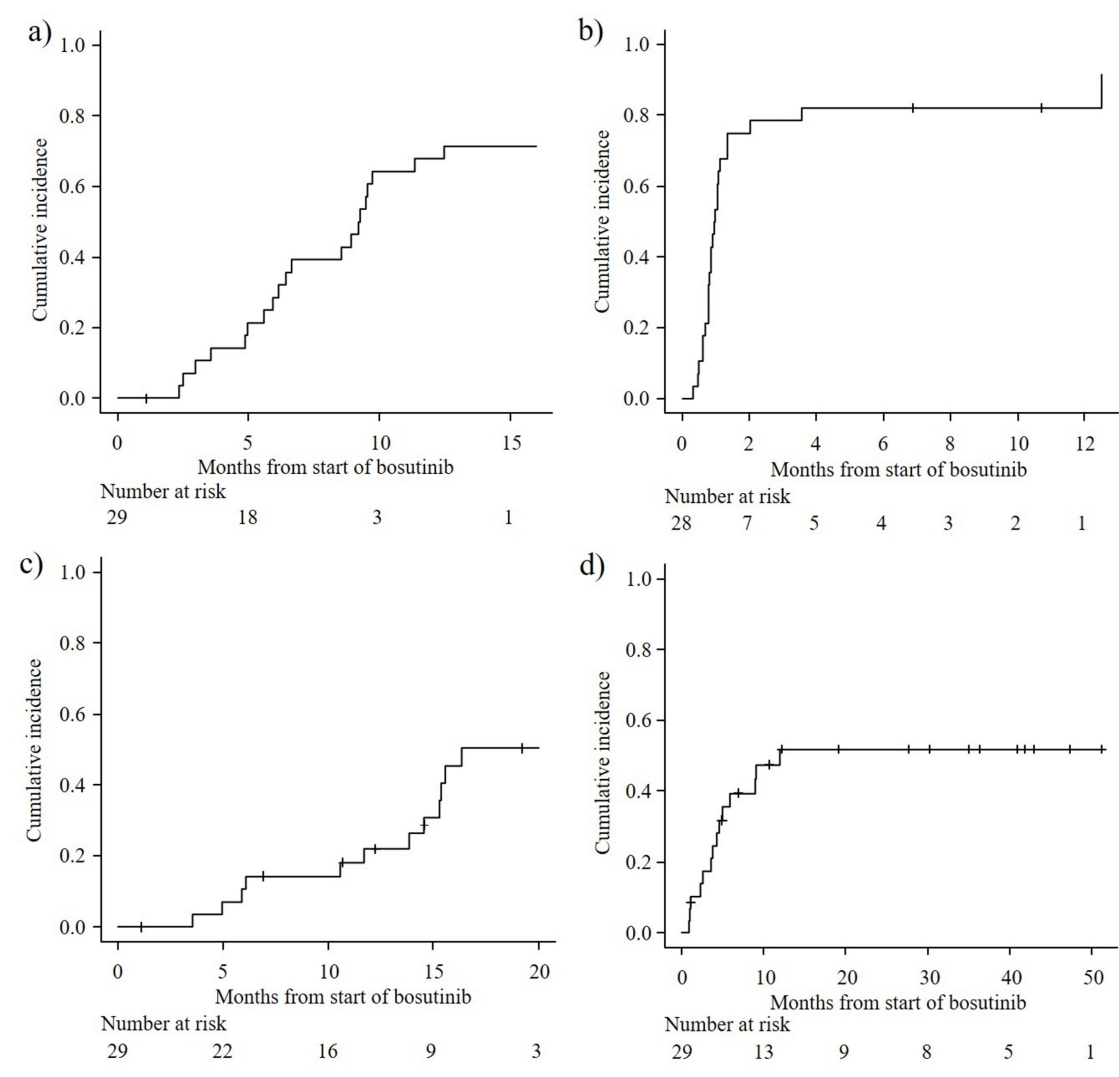
Cumulative incidence of major molecular response (MMR) (a), complete hematologic response (CHR) (b), MR4 (c), and treatment discontinuation (d). The cumulative incidence of MMR at 12 months is 67.9% (95% CI, 45.8–82.6%) (a). Cumulative incidence of CHR at 12 months is 82.1% (95%CI, 60.7-92.5%) (b), and MR4 at 12 months is 22.1% (95% CI, 8.7-39.4%) (c). The 24-month cumulative incidence of treatment discontinuation is 51.8% (95% CI, 34.5–71.7%) (d).

At a median follow-up of 23 (range, 1.1-51.9) months, the primary endpoint, the cumulative incidence of MMR at 12 months, was 67.9% (95% CI, 45.8-82.6%). The overall MMR rate during follow-up was 68.9% (Figure 1A). On exploratory analyses, the cumulative incidence of CHR at 12 months was 82.1% (95% CI, 60.7-92.5%), and the overall CHR rate was 85.7% (Figure 1B). The cumulative incidence of MR4 at 12 months was 22.1% (95% CI, 8.7-39.4%), with an overall MR4 rate of 48.2% during follow-up (Figure 1C). During follow-up, all patients remained alive, and the OS rate at 24 months was 100%. Neither progression to AP/BP nor loss of MMR was observed during follow-up.

Adverse events, treatment discontinuation, and subsequent treatment

Clinically relevant adverse events are summarized in Table 2, and detailed adverse events and treatment modification are shown in Table 3. Hematological toxicities, including anemia (65.5%) and thrombocytopenia (58.6%), were frequently observed but were generally manageable. Of non-hematological adverse events, increased alanine aminotransferase was the most frequent event, occurring in 24 patients (82.8%), including 13 (44.8%) with grade 3-4 elevation. Hepatic toxicity was the leading cause of treatment discontinuation, accounting for seven cases. Diarrhea occurred in eight patients (27.6%) and was grade 3 in one patient. Overall, 17 patients (58.6%) required dose reduction, and 14 (48.3%) required treatment interruption due to adverse events during therapy. The cumulative incidence of treatment discontinuation at 24 months was 51.8% (95% CI, 34.5-71.7%) (Figure 1D). Except for one patient who failed to achieve MMR and another who discontinued for financial reasons, all treatment discontinuations were due to adverse events. Of the 14 patients who discontinued bosutinib, all received subsequent TKI therapy. Dasatinib (n=6) and imatinib (n=5) were the most common second-line agents. Seven patients required third-line therapy, most commonly asciminib (n=4), and one patient proceeded to fourth-line treatment.

**Table 2:** Clinically relevant treatment-related adverse events (n=29) Adverse events were graded according to Common Terminology Criteria for Adverse Events (CTCAE) v5.0.

Adverse event	Any grade n (%)	Grades 3–4 n (%)
Anemia	19 (65.5)	3 (10.3)
Thrombocytopenia	17 (58.6)	2 (6.9)
Alanine aminotransferase increased	24 (82.8)	13 (44.8)
Diarrhea	8 (27.6)	1 (3.4)
Cardiac failure	2 (6.9)	2 (6.9)
Pleural effusion	3 (10.3)	0

**Table 3:** Treatment-related adverse events and treatment modification (n=29). Adverse events were graded according to Common Terminology Criteria for Adverse Events (CTCAE) v5.0. Counts represent numbers of patients, and percentages were calculated using all 29 treated patients as the denominator. Treatment modification columns list the number of patients in whom the adverse event contributed to dose reduction, interruption, or discontinuation; patients could contribute to more than one row.

System organ class / adverse event	Any grade n (%)	Grades 3–4 n (%)	Dose reduction (n patients)	Interruption (n patients)	Discontinuation (n patients)
Blood and lymphatic system disorders					
Leukopenia	4 (13.8)	1 (3.4)	0	0	0
Neutropenia	1 (3.4)	1 (3.4)	0	0	0
Anemia	19 (65.5)	3 (10.3)	0	0	0
Thrombocytopenia	17 (58.6)	2 (6.9)	1	1	0
Eosinophilia	1 (3.4)	0	0	0	1
Hepatobiliary disorders					
Alanine aminotransferase increased	24 (82.8)	13 (44.8)	9	8	7
Gamma-glutamyltransferase increased	17 (58.6)	2 (6.9)	0	0	0
Alkaline phosphatase increased	8 (27.6)	0	0	0	0
Pancreatic disorders					
Amylase increased	10 (34.5)	1 (3.4)	1	0	0
Lipase increased	1 (3.4)	1 (3.4)	0	0	0
Pancreatitis	1 (3.4)	0	0	0	1
Cholecystitis	1 (3.4)	1 (3.4)	0	0	1
Gastrointestinal disorders					
Diarrhea	8 (27.6)	1 (3.4)	4	2	0
Nausea	8 (27.6)	0	0	0	1
Abdominal pain	3 (10.3)	0	0	0	0
Enteritis	1 (3.4)	0	0	0	0
Mesenteric panniculitis	1 (3.4)	1 (3.4)	0	0	0
Anorexia	1 (3.4)	1 (3.4)	2	0	0
Cardiac disorders					
Cardiac failure	2 (6.9)	2 (6.9)	0	1	0
Angina pectoris	1 (3.4)	1 (3.4)	0	0	0
Myocardial ischemia	1 (3.4)	1 (3.4)	0	0	0
Respiratory, thoracic and mediastinal disorders					
Pleural effusion	3 (10.3)	0	3	0	0
Pericardial effusion	1 (3.4)	0	0	0	0
Infections and infestations					
Upper respiratory tract infection	2 (6.9)	0	0	0	0
COVID-19	1 (3.4)	0	0	0	0
Investigations					
Creatinine increased	7 (24.1)	0	0	0	0
Hyperkalemia	3 (10.3)	0	0	0	0
Hyperglycemia	2 (6.9)	0	0	0	0
Skin and subcutaneous tissue disorders					
Rash	9 (31.0)	0	1	0	0
General disorders and administration site conditions					
Fatigue	3 (10.3)	1 (3.4)	0	0	0
Pyrexia	3 (10.3)	0	0	3	0

## Discussion

In this multicenter, retrospective study, first-line bosutinib achieved a cumulative incidence of MMR at 12 months of 67.9%, consistent with the results reported in prospective clinical trials [7,9-11]. Despite an older median age and a high prevalence of comorbidities, the molecular response rates observed in our cohort were broadly consistent with those reported in clinical trials, suggesting that bosutinib remains effective in routine clinical practice. A notable finding of this study was the high frequency of dose modification. Most patients initiated bosutinib at a reduced dose and subsequently underwent step-up dose escalation, with 75.9% eventually reaching ≥400 mg. Step-up dose escalation from 100 mg has been reported to be beneficial in relapsed or refractory CML [17]. No treatment discontinuations were attributed to diarrhea in our cohort, suggesting that initiating bosutinib at reduced doses may also be associated with favorable gastrointestinal tolerability in the first-line setting.

Hepatic toxicity has been consistently recognized as an important adverse event associated with bosutinib. In the final analysis of the BFORE trial, liver function abnormalities were the most common adverse events leading to treatment discontinuation with bosutinib [9]. In a Japanese phase 2 study, liver function-related treatment-emergent adverse events were frequent, occurring in 80.0% of patients, with grade 3/4 events in 48.3% [10]. In the three-year follow-up of this study, discontinuation due to liver function-related adverse events occurred in 16.7% of patients, most commonly because of increased alanine aminotransferase (ALT) and/or aspartate aminotransferase (AST) [11]. Expert recommendations also emphasize frequent liver function monitoring during the early phase of bosutinib therapy, when liver enzyme elevations are most likely to occur, as well as individualized dose adjustment for managing bosutinib-associated adverse events [18]. The high frequency of ALT elevation and treatment discontinuation due to hepatic toxicity in our cohort is consistent with these observations and underscores the clinical importance of these management strategies. Previous studies in Asian populations have suggested that favorable outcomes can be achieved even at lower doses [19]. From this perspective, a more flexible dosing approach, rather than uniform escalation to the maximum dose, may help balance tolerability and efficacy in selected patients. However, this hypothesis should be interpreted cautiously and requires validation in prospective studies. This study has several limitations, including its retrospective design, small sample size, and limited follow-up duration. In addition, potential selection bias and heterogeneity in treatment approaches, including variability in dose adjustments and assessment intervals, may have influenced the observed outcomes. Furthermore, the lack of protocol-defined dosing strategies and assessment intervals may limit the reproducibility of the findings.

## Conclusions

First-line bosutinib demonstrated molecular response rates broadly consistent with those reported in clinical trials in patients with newly diagnosed CML. However, treatment discontinuation due to hepatic toxicity remains a clinical concern in real-world practice. Careful monitoring and individualized dose adjustment may help maintain treatment efficacy while improving tolerability.
